# Standardized Functions for Smartphone Applications: Examples from Maternal and Child Health

**DOI:** 10.1155/2012/973237

**Published:** 2012-12-13

**Authors:** Mary Jane Rotheram-Borus, Mark Tomlinson, Dallas Swendeman, Adabel Lee, Erynne Jones

**Affiliations:** ^1^Semel Institute for Neuroscience and Human Behaviour, University of California, Los Angeles, 10920 Wilshire Blvd., Suite 350, Los Angeles, CA 90024, USA; ^2^Department of Psychology, Wilcocks Building, Ryneveld Street, Stellenbosch 7600, South Africa

## Abstract

Millennium Development Goals (MDGs) are unlikely to be met in most low- and middle-income countries (LMIC). Smartphones and smartphone proxy systems using simpler phones, equipped with the capabilities to identify location/time and link to the web, are increasingly available and likely to provide an excellent platform to support healthcare self-management, delivery, quality, and supervision. Smart phones allow information to be delivered by voice, texts, pictures, and videos as well as be triggered by location and date. Prompts and reminders, as well as real-time monitoring, can improve quality of health care. We propose a three-tier model for designing platforms for both professional and paraprofessional health providers and families: (1) foundational functions (informing, training, monitoring, shaping, supporting, and linking to care); (2) content-specific targets (e.g., for MDG; developmentally related tasks); (3) local cultural adaptations (e.g., language). We utilize the Maternal and Child Health (MCH) MDG in order to demonstrate how the existing literature can be organized and leveraged on open-source platforms and provide examples using our own experience in Africa over the last 8 years.

## 1. Introduction 

The Millennium Development Goals (MDGs) of the World Health Organization (WHO) are unlikely to be met in most low- and middle-income countries (LMIC) [1, 2]. The rapid diffusion of mobile technologies over the last 10 years [3] creates opportunities for mobile phones to significantly contribute to achieving MDGs. LMIC have bypassed heavy investment in land-line infrastructure and focused instead on wireless mobile phone infrastructure. Mobile phones can be used by providers (physicians, nurses, administrators, lab technicians, or community health workers (CHW)) in hospitals, clinics, or on home visits for surveillance, monitoring health status, and easing emergency situations. For example, several studies show that training CHWs in rural areas via mobile phones can improve health conditions and alleviate financial burdens on understaffed hospitals [4, 5]. Concurrently, mobile phones may improve patients' access, quality, and utilization of care, as well as allow patients to receive health information, skills, support, and crisis services directly for a specific health condition. A standardized platform model could help mobile designers to leverage their individual applications to address MDGs—rather than replicating a common pattern of *siloed* or *categorical* mobile applications—that integrate programming for common functions and specific disease-related information that is culturally tailored.

We have arbitrarily selected Maternal and Child Health (MCH) MDGs as a prototype example for how to build on existing mobile applications and to expand and generate novel applications efficiently. MDG4 (reduce child mortality) and MDG5 (improve maternal health) are not likely to be met by most LMIC, even though most maternal and child deaths are caused by preventable or treatable conditions [1]. Maternal and child survival and health depend on scaling up solutions for common, preventable causes of morbidity and mortality. A recently published comprehensive literature review of mHealth on maternal and infant outcomes [6] focused specifically on prenatal and neonatal health within a developmental framework. In this paper, we expand this work and broaden the scope of their examination by providing a conceptual framework to guide development and analysis of applications that are emerging globally. 

## 2. Framework: Foundational Functions Content Tailoring, and Local Adaptation

**Figure 1:**
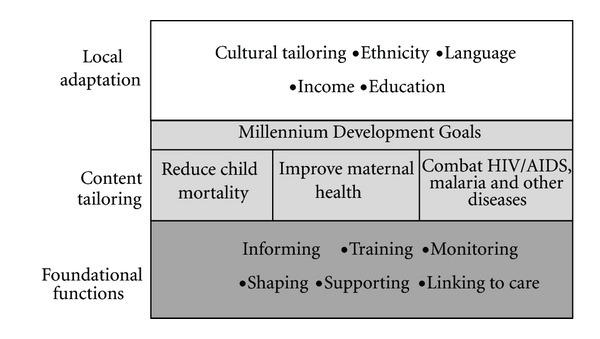
Framework for designing mHealth tools to achieve Millennium Development Goals for MCH.

A three-tier framework is proposed in Figure 1. A set of foundational functions are shared across many content domains. Regardless of training level or setting, effective healthcare providers demonstrate the following functions with patients: (1) provide information to be applied in daily life; (2) build skills; (3) monitor health status; (4) shape positive health behaviors; (5) create social networks that support change; and (6) address environmental barriers (e.g., transport to hospitals) [7]. These tasks can be facilitated by using mobile phones for a specific activity, or by a system of applications that are able to inform, train, monitor, shape, support, or link providers or patients in need of services for a specific health outcome. 

The framework's second tier is the synthesized knowledge on specific content areas. The foundational functions are necessary and useful to a range of specific health goals: diabetes, obesity, premature births, high blood pressure, HIV, hepatitis, TB, and many other disorders. However, each of these diseases and conditions has a set of protective health behaviours to be implemented. For example, all patients with diabetes should implement changes in diet, exercise, monitor blood sugar and Hba1c, and visit a health provider routinely. The same challenges exist in Africa, Asia, and the United States. In this paper, we are focusing on MDG 4 and 5 which can be addressed by a limited set of robust scientific kernels of information, skills, and support that are applicable across regions, populations, and languages. 

Finally, the third level is the freedom to adapt and tailor applications for a specific population, language, or region. When the robust elements have been identified at the functional level and the synthesized knowledge has been identified for a specific content area, communities can then identify the aspects of an application to adapt and customize to their local conditions and cultural contexts. The ways in which tailoring is feasible become clear when the robust elements have been defined. 

## 3. Smartphones and Smart Proxy Systems

The three-tier model may potentially guide the development of open-source platforms for mobile designers, interventionists, and public health specialists that allow the field to manage knowledge and applications more efficiently. We focus on smartphones and smart proxy systems for two uses: improving the quality of care by healthcare providers and improving families' direct access to health information and support services. Although we focus on the needs of pregnant women and families as an example, the opportunities and challenges are similar for a broad range of health conditions. 

There are more than 5.3 billion mobile phone users globally [3]. By 2012, more than 50% of these mobile phones are anticipated to be equipped with global positioning systems (GPS) and web-connectivity (i.e., “smartphones”). Smartphones are not only represented by current cutting edge and expensive devices (e.g., iPhones); simpler phones that are currently diffused globally can be “smart proxies” when linked to information management systems via the web through simpler operating systems, SMS (i.e., text message) systems, or interactive voice response (IVR) systems [8, 9]. Thus, smartphones are defined here as having the capacity to link to information management systems, while also providing real-time location data based on GPS, radio tower triangulation, or user input of postal codes, for example, if not equipped with GPS [10]. The location-aware capabilities of mobile phones, while not critical to many of the examples we provide below, are a key functionality that can be leveraged to link patients to health information, resources, and interventions in their local community. To meet many of the MDGs, strategies must focus on service system strengthening: access, referral, coordination, efficiency, and policies [11, 12]. Equally important, the broad availability of these devices offers the opportunity to scale prevention and care to an unprecedented magnitude at small marginal cost [10].

## 4. Targeted Users: Healthcare Workers and Families

Mobile technologies are particularly useful to healthcare providers. There were at least 2.3 million physicians, nurses, and social workers needed globally in 2006 [13]. Today, India alone needs 2.4 million nurses [14] and China needs another 5 million nurses [15]. This large unmet need for healthcare professionals will not be met before the year 2050 [13]. Today, there are more than 40 million paraprofessional community health workers (CHWs) who are shifting tasks from professional healthcare providers to less expensive and less well-trained personnel [16, 17].

Mobile phones are useful to the full range of providers, but perhaps are most important for those with the least training. Mobile phones offer the opportunity to supplement interpersonal interactions with videos, real-time support, quality of care monitoring with location and time-stamps, and linkage to more experienced health personnel. Thus, linkage and coordination of care, particularly in rural settings with few healthcare personnel, may best be served and networked with mobile technologies. 

The second major set of users is families. Mobile technologies also offer the opportunity to support families directly in managing their own health, while linking their self-management efforts to healthcare providers and other resources. Several review papers have identified randomized trials examining the efficacy of text message or voice call interventions for health education, reminders, monitoring, or management of medications and symptoms of asthma, smoking, diabetes, and HIV [18–21]. However, most of the existing projects typically rely only on the most basic text message or voice call functions of mobile phones and do not leverage the enhanced capacities of smart-phones or smartphone proxy systems.

We conducted a literature review that updates and expands the Tamrat and Kachnowski review [6]. To identify mHealth applications for maternal and child health, the current literature review was conducted in March 2012, using searches on Google, Google Scholar, and PubMed. Search terms included combinations of the following keywords: maternal, maternal health, public health, child, maternal mortality, infants, infant health, mobile, mHealth, mobile health, midwife, midwives, maternal mortality, maternal morbidity, cell phone, cellular phone, community workers, and community health worker(s).  Table 1 summarizes the studies that utilize mobile technology to support healthcare. We list studies by target user (healthcare provider, families, or both) and identify the mobile phone function—informing, training, monitoring, shaping, supporting, and/or linking. We identified 33 studies: 13 target families, 12 target providers, and eight target both families and providers. 

## 5. Foundational Functions: Overview

Evidence-based interventions (EBIs) for a range of MCH challenges share common functions, processes, and elements. Knowledge management is a key challenge in the adaptation and diffusion of EBI for MCH [22]. Rather than focusing on a manual that includes the common functions, as well as the unique issues to a specific EBI, we are proposing that there is a set of common functions across all mobile applications for MCH that, if underlying new applications, would reflect the robust theoretical and programmatic components that support efficacy and cost efficiency. Typically, meta-analyses, research syntheses, Delphi panels, and novel theories have been the traditional strategies for identifying robust EBI features. This approach has been used in other areas. For example, seven foundational practices are common to 80% of evidence-based child mental health programs for depression and anxiety (exposure, cognitive restructuring, child psychoeducation, relaxation, modeling, parent psychoeducation, and self-monitoring) [23]. 

Smartphones can facilitate broadly scalable delivery of six foundational functions for effective prevention and treatment interventions:

1. *informing* about health risks, healthy behaviours, and available resources;
2. *training* new behaviours by providing text messages, calls, pictures, or even videos that model the desired behaviours;
3. *monitoring* behaviour in real time, including unobtrusive and automatic monitoring to reduce the burden of self-monitoring;
4. *shaping* healthy behaviours through monitoring, feedback, prompts and reminders, encouragement, and rewards provided in real time;
5. *supporting* development and maintenance of healthy behavioural routines by linking to peers, friends, family, or healthcare workers for social support and instrumental support services;
6. *linking* to healthcare or results from diagnostics tests.

Many mobile applications for different functions and different content areas use idiopathic programming languages and structures. Parallel to the standardization that made the internet scale globally, mobile applications for public health need open source programming and standardization. 

## 6. Content Tailoring

MCH faces different challenges in different regions of the world. In order to meet the MCH MDGs for pregnant women in LMIC, healthcare providers must address the most prevalent diseases in a specific region. For example, in South Africa, HIV, TB, malnutrition, alcohol abuse, and depression are significant health challenges that impact at least 77% of pregnant women, often concurrently [24]. In Indonesia, pregnant women are most vulnerable to a lack of clean water and toilets and smoking among family members. The specific risks vary in different countries; however, there are a limited number of major challenges in each region. Three major domains influence MCH MDGs: reduce infant mortality, improve maternal health, and reduce infectious diseases (HIV, malaria, TB). Smart-phones have the potential to facilitate provider training, monitoring, real-time support, and link providers and the families they serve to healthcare services. 

There are also common robust features of the same MCH challenges in different regions. For example, mothers must be aware that alcohol use during pregnancy causes long-term developmental challenges for children. The features of fetal alcohol syndrome (FAS) are similar across geographic region and culture: facial deformities, low birth weights, and cognitive deficits [25]. The amount of alcohol that causes deficits is a highly relevant dimension. However, there are regional variations in the size of containers for drinking (i.e., amount consumed), types of liquor consumed, and stigma that children may experience for exhibiting FAS symptoms. 

Therefore, in addition to the standardized functions, it is necessary to synthesize and build on the research evidence for each specific maternal or infant outcome. Synthesized knowledge of the common content in a domain facilitates the development of mobile applications for the robust components for each content area. Our ability to synthesize and organize knowledge in ways that are simple, robust, and broadly available is our current challenge. 

## 7. Local Adaptation

Community-based organizations have long argued that EBIs are insensitive to the local contexts. Specific ethnic groups, local customs, or context-specific situations have challenged designers of EBI to specify the robust elements or key characteristics of each EBI. There has been great variability in the definition of these elements and characteristics. The proposed three-tier structure is used as a framework for deconstructing an EBI to identify the key foundational functions and the content issues addressed. Once the functions and content-based knowledge are known, the provider or family may adapt the EBI to their local conditions. Examples of how these levels merge are outlined below. 

## 8. Foundational Functions Applied to MCH MDG

### 8.1. Informing

The foundational skills are represented in a broad range of intervention tools: text messaging, video chats, YouTube videos, and GPS systems linked to web-based data, for example. To date, the function of informing most typically uses text messages and voice calls. Text messaging is universally available on all mobile phones, while pictures, video and recording capabilities are becoming increasingly available. Text messages can deliver specific information about health risks (e.g., risks of alcohol) and preventive behaviours (e.g., ways to protect infants from HIV) and can provide referrals and motivational “tips” [26]. This approach is currently being disseminated broadly in the United States in the “text4baby” [27] program, in which a mother (or father or other family member) simply texts “Baby” to 511411 and then immediately receives prompts to enter the baby's expected due date and postal code to confirm participation in the program. The program then sends several free text messages each week (even for those without a text messaging service plan) in English or Spanish that are tailored to the baby's age (e.g., importance of getting immunizations, information on breastfeeding). Each message also includes toll-free telephone numbers to link to free and low-cost services associated with the message topic and tailored to the mother's location (via entry of the postal code). 

Written information is not likely to be useful for many populations with limited education; however, automated voice messages can provide the same function as text messages in a more broadly understandable medium. Interactive voice response (IVR) systems are particularly engaging, especially for people with limited literacy. For example, an IVR system was recently demonstrated in Bangladesh in which simple informational me ssages, such as the importance of clinician-assisted birth or hand washing, are delivered in a brief soap opera format that builds the storyline over each successive call [28]. In addition, the IVR system includes a quiz at the end of each call, which verifies receipt of the information, reinforces learning through the cognitive benefits of testing [29], and provides an incentive for participation with free mobile airtime sent to the user when correct answers are provided. The system even allows multiple users to share one phone, while each user participates in the program individually. For example, multiple mothers in a family or village may share a family phone or a phone “rented” from a village phone operator, but paid for with airtime provided by the program.

As “smarter” phones with picture and video capabilities become increasingly affordable, an even more engaging medium becomes available to provide detailed health information. With minimal monetary and time investment, existing informational videos on the subject of prenatal care, for example, could be mined from the internet for broad dissemination via mobile phones. 

Information dissemination by mobile phone is deployed on a large scale with the MoTeCH system in Ghana. Built as a “plug-and-play” system with open-source programming that can be adapted to many languages, the MoTeCH program is used to diffuse information to pregnant women about risk factors, set expectations for normal deliveries, and identify local obstetrical resources [30]. The information system is designed in modules so that different risk factors can be adapted to different regions of the world, depending on the needs in the local community. Not only is this example impactful in its current application, the open-source and plug-and-play aspects of the programming are critical in future investments of mobile technologies for improving MCH. However, while MoTeCH is intended to be broadly diffused to other health providers and research teams in other countries, trying to reprogram the initial MoTeCH program to an open-source system has required more than 18 months to date. If standards had been established prior to its implementation, the program would be ready for broad diffusion.

Resources that are difficult to locate in LMIC (e.g., emergency physical and mental health support) can be more easily identified using the location-aware functionality of GPS-enabled smartphones and similar proxy systems (e.g., radio tower triangulation) to facilitate access to and delivery of information that is relevant to the location of the family. This data is often not available for primary healthcare services, community-based organizations (CBO), or emergency physical and mental health support. Especially in LMIC, the location of the closest health center is often unclear. For example, scanning the web, Alcoholics Anonymous (AA) reports that there are at least 50 active and established groups in Cape Town, South Africa. Yet, in interviews with over 2000 families, providers in ten clinics, and health administrators in the townships, no one had ever heard of AA [31, 32]. 

### 8.2. Training

Training for health providers must be a continuous process if the quality of care is to improve over time. Information is necessary, but often not sufficient, to change behaviours [33–35]. While text and voice communication can be sufficient to train many new behaviours, smartphone pictures and videos can demonstrate positive role models performing complex behaviours. In our collaborative research in Cape Town, CHWs were trained using 22 videos that captured common risk issues that CHWs often confront in the home: mothers who abuse alcohol, malnourished children, and HIV seropositivity undisclosed to family members. These videos are used in initial training, but could be delivered on smartphones to provide boosters to CHWs over time. Trainees can also send back short videos and brief questionnaire responses regarding the newly learned skill so that the quality of training and mastery of new behaviours may be monitored [36], and checklists and homework can be automatically downloaded, answered, and uploaded at specific times during training [26]. Training boosters or updates can also ensure continued learning and quality of care over time. In many LMIC, CHWs often have limited literacy; the video capabilities of smartphones facilitate training, support, and boosters. In light of recent concerns about the number of tasks that CHWs are being required to perform, such training will prove invaluable.

Training is especially critical when complex decision making is needed to determine appropriate medical regimens. For example, guidelines for treatment of child malnutrition require calculating target weights and correct dosages. In addition to supporting the ability to track patient information over time, D-tree [37] created interactive mobile software to assist health workers in making these calculations and guide them through the process of screening, examination, counselling, and treatment.

### 8.3. Monitoring

 Self-monitoring is a key strategy for supporting behaviour change [38, 39]. It is essential for informing and training, and it provides timely and relevant feedback for behavioural shaping and support, either for providers or families [40]. Mobile phones can be utilized for patient self-monitoring of symptoms, behaviours, and attitudes [41]; CHW monitoring of patients; and monitoring of provider practices in healthcare delivery. Monitoring and self-monitoring via mobile phones has been demonstrated to be acceptable, valid, and reliable for a variety of populations and behaviours, including physical activity [42], diet [43], and blood glucose in children [44]. Evidence suggests that recording a given behaviour alters the behaviour up to 15% [45]. Smart-phones also allow many behaviours to be recorded unobtrusively and automatically over time. For example, accelerometers are being built into phones and developers are designing new applications to expand utilization of this feature. 

Smart-phones enable more accurate and precise self-monitoring, for example, by prompting users to record an image, text, or audio clip immediately when a behaviour occurs. Prompts in real time also allow automatic storing of geo-tagged and time-stamped entries in a personal database. Spatio-temporal traces of behaviours throughout a day, week, or month can tremendously increase an individual's self-understanding. In addition, these traces can increase clinician or supervisor knowledge of detailed behavioural patterns [36].

In South Africa, we use mobile phones to monitor where and when CHWs are delivering care, as well as the topics addressed in each client encounter [46]. This type of monitoring significantly improves the quality of delivery by repeatedly prompting CHWs and enhancing decision-making support in supervisory meetings. CHWs can also be prompted to cover particular content for that day (before entering the home), report the exit from a home, and probe for the context and success of the visit. Automated probes can also be sent to the mother's phone inquiring about the content and quality of the interaction, providing real-time data that may corroborate the CHW's report.

Furthermore, innovations in peripheral biosensors that can be linked to mobile phones (often wirelessly) show tremendous promise in assessing and monitoring health status. For example, Microsoft is supporting the development of a smart-phone-based fetal monitoring device that addresses the challenge of lack of access to prenatal care via a low-cost Doppler-based ultrasound device that connects to a smart-phone and enables mothers to track and record fetal heart rate and activity (i.e., kicks per minute) while automatically transmitting the data to healthcare providers at a district hospital [47]. Ozcan is devising small microscope attachments for mobile phones that can diagnose malaria and perform CD4 counts [48]. Such monitoring devices can be used not only by mothers or consumers, but also by providers, especially those in rural settings who lack access to high-quality laboratories. 

The number of mobile applications for self-monitoring rise exponentially for persons in middle- and high-income countries. Healthy mobile phone users can utilize these applications routinely or when acquiring a new habit. Unfortunately, these applications are not linked to electronic medical records and thus are not easily accessible by providers who could use the information to better manage health care. 

### 8.4. Shaping

Once new behavioural skills are learned, they must be integrated into a person's daily routine. Smart phones and smart proxy systems deliver prompts, reminders, alarms, warnings, and supportive messages to shape these new behaviors. Text-messaging systems have already proven effective for helping people to adhere to medication schedules [4], stop smoking [49], increase exercise [26], and manage diabetes [19]. Cell-Life [50] uses a series of text messages, sent over a period of 10 weeks, to prompt HIV+ mothers to bring their infants to clinic appointments and for HIV testing. Although data collection is still in progress, initial findings support the effectiveness of the intervention, with mothers in the intervention condition (i.e., receiving text message reminders and information) being more likely to bring their infants to the 6-week HIV testing and to view the text messages as supportive and helpful. Weekly SMS “goal checks” help mothers assess whether health behaviour goals are being met, using tailored responses. Prompts or reminders, which can be triggered at times and/or locations informed by the user's monitoring data and goals, can successively shape behaviours with continuous feedback on progress [51].

There is a process of successive approximation towards goal setting that requires constant recalibration of the goals, expectations, and factors monitored to indicate positive outcomes. Smartphones can also deliver rewards when the desired behaviours have occurred. The possible reward modalities are ever-expanding to include mobile airtime credit, praise messages, music, videos, social rewards from friends and family [52], or virtual rewards like Intel's Ubifit application that provides a flower garden screen saver which colourfully grows as the user nears the physical activity goal [53].

### 8.5. Supporting

Smart-phones can facilitate obtaining the instrumental and social support needed to sustain healthy behaviours or effective service delivery over time. For example, CHWs in Gambia have recommended that mobile phone systems could support coordination of ambulance transportation when transferring patients, to prevent maternal and fetal mortality [54]. In Thailand, a text-messaging system linked to electronic medical records and an immunization registry is currently being tested to increase immunization rates among vulnerable pregnant women and children [55].

Smartphones are a vehicle for integrating individuals into their social contexts as a source of support. Mobile videos and teleconferencing have been used to link mentors with mothers to support them in making incremental behavioural modifications through goal-setting and problem-solving [56]. Social and emotional support from peers may help mothers with parenting challenges when support may not be readily available from family and friends. For example, postnatal mothers and their preferred social support person were both targets of a text message intervention to increase postnatal physical activity [51]. Improved health outcomes are directly related to strong and supportive relationships, while poor health outcomes and increased mortality are common risks of those who are socially isolated and lack a sense of belonging. Meet-up functions have been used to organize huge gatherings, not only in high- and middle-income countries, but also for political purposes in LMIC. Drop-in meet-ups could be the new alternative to scheduled counseling groups in the future. 

In South Africa, we programmed diabetic women's phones to link to a randomly assigned peer, a “Diabetes Buddy” [57]. Diabetes Buddies exchanged text messages to support each other to exercise, monitor their blood glucose, and eat healthy meals over a 6 month period. As a small pilot study, we found that about 1/3 of the randomly assigned dyads were successful, 1/3 texted or contacted each other at a low rate, and 1/3 appeared supportive, but the Buddy was not the key source of support. The women who utilized the support system the most were those who had the most uncontrolled diabetic status and were the most disabled. In this program, the support functions of mobile phones supplemented an in-person intervention program to change daily routines. 

Not only do mothers need support, but CHWs are often emotionally overwhelmed by the difficult circumstances that they encounter on their home visits. By using a smartphone to access support, CHWs are empowered to deal with some of these complex issues, as it is not uncommon for CHWs to encounter patients suffering from physical and emotional abuse, poverty, and illness [46]. The support of peers in the workforce can be critical ingredients to a well-functioning program. Burnout can be alleviated if there is a norm to take breaks for relief, resolve conflicts quickly, and access support routinely. If CHWs are to acquire the level of efficacy that is consistently demonstrated in the small efficacy trials, there will need to be routine systems for providing support. The use of smartphones and smart proxy systems will be fundamental for delivering support to both mothers and providers in LMIC [46].

### 8.6. Linking to Care

An integrated healthcare system provides the most effective and efficient care. MoTeCH not only provides informational voice messages, but it also alerts care providers to specific cases that require additional followup. For example, a text message alert can be sent to a nurse if a mother misses her clinic appointment. Thus, mobile technology can be used universally, as well as at a more targeted and need-specific level of care. 

Thaddeus and Maine provide a framework for understanding the impact of delay in seeking and obtaining medical care on the high rates of maternal mortality and obstetric complications in developing countries [58]. Mobile technology can directly address these delays and has significant potential to decrease rates of mortality and complications by providing psychoeducation about symptoms, facilitating transportation, and coordinating care to the appropriate facility. 

For example, qualitative evidence from MoTeCH pilot programs in India demonstrated that 24-hour obstetric mobile phone-based helplines mitigated delays associated with deciding when to seek medical care, identifying appropriate health facilities, and receiving treatment. The Clinton Health Access Initiative [59] found that even when infants were tested for HIV, parents frequently did not receive test results and did not receive them in a timely manner, which impacted effective treatment and PMTCT loss to follow-up rates. The use of SMS-driven printers that link laboratories and community clinics via GSM mobile networks have decreased the turnaround time for test results by more than half. 

## 9. Discussion

Our goal is to increase awareness and investment in marginally low-cost, broadly-accessible smartphone systems as a vehicle for global and scalable healthcare innovations targeting healthcare prevention and treatment. The continuous connectivity between individuals, their mobile phones, and the web creates opportunities for improving health for individuals, families, and communities. Concurrently linking three information sources—phone location traces over time; individual ratings of thoughts, feelings, and actions; web-based data sets (of a pregnant mother, CHWs, or healthcare provider)—can foster personal insight and behaviour change, and transform scientific research and training. Moreover, this information is available at very low marginal effort and cost, increasing the feasibility of global deployment and impact. Paralleling the web's explosion of cheap, accessible public information, mobile phones offer an unprecedented increase in access to documenting and providing feedback to ourselves or our healthcare providers about our daily lives. 

The information available is of much higher quality, validity, and reliability than any information previously available, and can include more diverse, specific, sensitive and scalable data types. When our mobile phones eventually become equipped with biosensors (e.g., point-of-care diagnostics), another exponential expansion of personal information will occur. Investing in the software and methodological infrastructure to support a broad range of public health innovations will allow us to harness the potential for improving our ability to meet the MDG.

The potential range of behaviours, diseases, and conditions that mobile personal sensing can address is broad, as are the diverse needs and users of the mobile to web infrastructure. The ease with which mobile applications can be programmed and updated creates tremendous opportunities for relatively quick, broad, and inexpensive diffusion of health behaviour innovations. This scalable technology can also be deployed affordably and effectively to revolutionize the health systems in developing countries, where the penetration rate of mobile phones is higher than traditional wired communications [3].

We propose that mobile phone technology can effectively support six standardized functions of complex interventions to promote Maternal and Child Health Millennium Development Goals. Specifically, smartphones and smart-phone proxy systems can be used to *inform* mothers about pregnancy-related healthcare tasks, resources, or warning signs via text message, automated voice message, interactive voice response (IVR) systems, or videos. Smart phones can be used to support the *training* of healthcare providers by using videos to model complex behaviours or by using text and other media to provide step-by-step instructions for new tasks. Mobile phones can also be utilized by patients to *monitor* their symptoms and behaviours [42], by CHWs to monitor patients, and by supervisors to monitor CHWs practices in healthcare delivery. Then, once behaviours have been learned, smartphones can help *shape* behaviours with reinforcements. Smart phones can also help provide emotional and instrumental *support* to both mothers and CHWs via connections with peers and healthcare services. Finally, smart phones can provide direct *linkage* to hospitals and clinics or emergency services.

We urgently need to improve access to quality health care. With healthcare budgets of under $30 USD per person in 33 of 41 African countries (70%) [60], low-cost platforms for transmitting health information, skills, support, and crisis services must be identified and developed. Access must be available to all members and participants of healthcare systems (doctors, nurses, community health workers, receptionists, drivers, and supply clerks) as well as to the patients and communities. Mobile technologies are one potential platform to facilitate achieving the MCH MDG. 

## 10. Challenges and Limitations to Be Addressed to Realize Smartphones Potential for MCH

Before we can achieve the vision for smartphones to broadly improve MCH, there are significant challenges that need to be addressed. First, *privacy* concerns and ethical safeguards for the access, utilization, and sharing of personal data are crucial. Procedures to protect personal health data, similar to those currently used with electronic medical records, need to be integrated with any electronic health system. Structural and procedural mechanisms that will enable us to address the simultaneous needs of scalable personalized healthcare and personal privacy accountability include firewall, encryption, and password protections on computers, phones, and web-based systems; data-sharing policies and tiered access for different user groups such as field workers and supervisors; creation of an audit trail; and ongoing monitoring of security threats. Given these protections, there is evidence that electronic records are, in fact, more secure than paper records and that many patients prefer electronic records. For example, Curioso [61] found that sex worker patients highly preferred the mHealth system when rolled out in their clinic because they were more concerned about privacy violations from their paper charts being left on desks, while the electronic records were perceived to be more secure.

Second, attention and sensitivity to cultural issues and local contexts can be critical in the effectiveness of mobile phone technology implementation. For example, a feasibility study in Sierra Leone [62] found that individuals often share a single phone with other family members, or even with neighbours, due to limited resources. Some women, when sharing phones with their husbands, must ask for permission to use the phone, limiting privacy and the type of health information that women found acceptable to be communicated by healthcare providers via mobile phone. In some instances, husbands became jealous and physically abusive if they suspected that the text messages that their wives were receiving were evidence of infidelity. Thus, awareness of the impact of local customs, practices, and resources on the use of technology (and healthcare decision making) should inform implementation design. 

Third, significant investment of resources in developing and integrating current technology into a cohesive, standardized, yet flexible system is needed. To date, there has not been a concerted effort in creating standardized interfaces, data collection strategies, or programming platforms. *Innovative user interfaces* are needed to make data collection as easy as using an iPod and as seamless and clear as using a thermometer. *Data analytics and visualization*, with the possibility of integration over multiple time points and varying levels of data aggregation, require further innovation and research around human-computer interfaces. An *open platform* (i.e., similar to the web) with well-defined, standard interfaces must be defined early, and in detail, in order to develop a broad architectural framework of interoperable and portable services, rather than individual stovepipes based on proprietary solutions [63]. In an initial review of websites that report federally funded studies, a University of Maryland researcher counted 486 mobile phone projects, with only 29 of those being actual mobile health interventions [64]. There are now hundreds of pilot programs on mobile health applications with numbers and investment increasing rapidly over the past several years. While the cumulative investment is substantial, each project recreates about 80% of the programming with different programming languages, interfaces, and is highly tailored to the specific research study [63]. The ubiquitous distribution of mobile phones, the relatively inexpensive costs for programming adaptions to open-source platforms (on the order of $50,000 USD in our experience with several developers and projects), and their massive scalability, all support great potential for cost-effective returns on investment.

Further, most mobile health demonstrations have not *integrated mobile data with web-based data sets*, which would allow us to infer activities and monitor environmental hazards or exposures—a huge leap in the types and scope of inferences possible by linking personal GPS date to Geographic Information System (GIS) data. Finally, the feature set implemented for these mobile health projects have not been able to take *full advantage of smartphone capabilities*. Investing now in the software and methodological infrastructure will allow us to harness the potential of smartphones by increasing the efficiency and scalability for healthcare delivery, and our ability to meet the MDG, over the long-term. 

The development of standardized function programming will facilitate cost-efficient creation of optimal strategies for capturing and sharing data for different types of information, diseases, and purposes (e.g., epidemiological observations, prevention, treatment, and self-management). Finally, the ease with which mobile applications can be programmed and updated creates tremendous opportunities for relatively quick, broad, and inexpensive diffusion of health behaviour innovations. 

Given these challenges and the current national and global climate to harness science and technology to improve public health, we encourage substantial investment to create mobile health platforms that serve the public good, by promoting health service innovations while attending to the need for the individual to control access and sharing of their personal data stream. The widespread wireless mobile phone infrastructure in LMIC is an untapped network that can facilitate low-cost, scalable delivery of, and access to, healthcare via smartphones and smart proxy systems to achieve Millennium Development Goals.

## Figures and Tables

**Table 1:** mHealth applications for maternal and child health by foundational functions.

Program	Description	Function					
		Inform	Train	Monitor	Shape	Support	Link
Target group: mothers/families only							

text4baby [27] United States Sponsor: Johnson & Johnson Outcome Data: Y	Family member texts “Baby” to 511411 and receives prompts to enter the baby's expected due date and postal code. Program sends several free text messages each week tailored to the baby's stage of development and toll-free telephone numbers to link to free and low-cost services associated with message topic based on mother's location.	×					×

Diabetes Buddies [57] South Africa Sponsor: University of California/University of Western Cape/Women for Peace Outcome Data: Y	Twelve-session curriculum, with weekly sessions on nutrition, exercise, disease self-management, managing negative emotions, and coping with stress. Participants used mobile phones to call and text one another. Project leaders sent daily texts to provide nutrition guidance and to prompt participants to exercise.	×		×	×	×	

Wired Mothers [65] Zanzibar, Tanzania Sponsor: DANIDA Outcome Data: Y	Used to link pregnant women to healthcare system via mobile phones, increase accountability for clinic attendance, provide educational SMS, and access to emergency services.	×		×	×	×	×

Better Border and Healthcare Program [66] Thai-Myanmar border Sponsor: Microsoft Research Outcome Data: Y	Automated SMS-based reminders for prenatal care visits. Combined web-based and mobile technology to generate appointments for healthcare personnel performing home visits to cross check, identify, and update the mother and child health indicators at healthcare facilities and household locations.			×	×	×	×

Baby is on its way [67] Serbia Sponsor: Ministry of Health/Medicomark Outcome Data: N	SMS messages corresponding to the mothers' stage of pregnancy with health support.	×				×	

CELLPHONES4HIV [68] South Africa Sponsor: CellLife/Vodacom/ USAID/PEPFAR/Raith/ Johns Hopkins/Right to Care Outcome Data: Y	10-week program with bulk-delivered SMS messages for prevention and treatment of HIV/AIDS. Information is provided on health education, linkage to care, increasing “treatment literacy.”	×			×	×	×

Project Masihambisane [69] South Africa Sponsor: UCLA/HSRC/NIH Outcome Data: Y	Women with HIV invited to attend four antenatal and postnatal small group sessions. Mobile phones used to collect and upload numeric, voice, and text-based data.			×			

OG Miner [70] South Africa Sponsor: nexGIN RC Outcome Data: Y	Data mining system that provides accurate classification of pregnant women at high risk for obstetrical complications.	×		×		×	×

IVR mobile phone quizzes [71] Bangladesh Sponsor: BRAC/Dimagi/World Bank Outcome Data: N	Short audio courses with interactive quizzes covering health information such as clinician-assisted birth, proper hand-washing techniques, and HIV-transmission knowledge. Courses build on one another in a soap-opera format with recurring characters. Passing a quiz at the end of the course is incentivized by free airtime delivered to the caller's phone.	×			×		

Safe Motherhood Project [72] Senegal Sponsor: WAHA International Outcome Data: N	Use of donated prepaid mobile phones to allow mothers to contact hospitals for referral during delivery, coupled with motorbike ambulances to transport women to these services.						×

ChildCount [73] Kenya Sponsor: UNICEF Outcome Data: Y	Pilot program using SMS messaging to help community health workers monitor child health outcomes.			×	×	×	×

Phones for Health [74] Rwanda Sponsor: GSMA Outcome Data: N	Mobile phone system linking community health workers to HIV patients. Allowed workers to collect and retrieve patient information.			×		×	×

Manoshi [75] Bangladesh Sponsor: BRAC/Gates Foundation Outcome Data: N	Project aimed to decrease death and illness among infants and mothers in urban slums of Bangladesh. Allowed community health workers to upload data from field areas to central database to link with doctors for specific medical advice.	×		×			×

Target group: providers only							

Philani Paraprofessionals [76] South Africa Sponsor: UCLA/Philani Program/Stellenbosch University/NIH Outcome Data: Y	Community health workers used mobile phones to collect data, monitor and support intervention delivery, and increase accountability through geo-timestamps.			×		×	

RESCUER [77] Uganda Sponsor: World Bank Outcome Data: Y	The RESCUER project was designed to link the traditional rural community health providers with the formal health delivery system, such that when an obstetric emergency occurs in a village, a traditional birth attendant calls for assistance from the nearest health unit.					×	×

HealthLine [78] Pakistan Sponsor: Microsoft Research Outcome Data: Y	Speech-based telephone system targeted towards CHWs who have low levels of literacy. Provides CHWs with important health information, using spoken prompts, to support home visits.	×	×			×	

Mobile Midwives [79] Aceh Besar, Indonesia Sponsor: World Vision Outcome Data: Y	Midwives provided with mobile phones and phone credit to link with healthcare systems. Outcomes include increased accuracy of data collection and an increase of midwives linking with health centers for professional advice.	×	×	×		×	×

Cell-PREVEN [80] Ucayali, Peru Sponsor: UN Foundation/Vodafone Foundation Outcome Data: Y	Health workers in a remote geographical area were given basic mobile phones to collect and report data to a centralized database using an interactive voice response system.			×		×	×

Ca:Sh [81] Haryana, India Sponsor: Dimagi/All India Institutes of Medical Science Outcome Data: Y	Mobile phones used for data collection on immunization, demographic changes, and prenatal care. Electronic health records made available via mobile phone.			×		×	×

Sisu Samrakshak [82] Andhra Pradesh, India Sponsor: UNICEF Outcome Data: Y	Mobile technology used to monitor nutrition and maternal/child health.			×			

SMS2Printer [83] Mozambique Sponsor: Clinton Health Access Initiative Outcome Data: N	System designed to accelerate return of results by allowing labs to print any test results from health centers with network coverage. Expanded program from initial successful pilot program.	×		×			×

Rwanda RapidSMS [84] Rwanda Sponsor: UNICEF/WHO Outcome Data: Y	Tools on mobile devices helped CHWs track pregnant women's progress, identify risk factors, monitor antenatal care, and communicate with clinics and hospitals to improve emergency response times.	×	×	×	×	×	×

Health at Home/Kenya [85] Kenya Sponsor: AMPATH Outcome Data: N	Data collection tool for collecting health outcome data, recording test results, and HIV counselling and testing. Uses GPS technology to identify family's location and allows quicker data transmission.			×		×	×

CycleTel [86] India Sponsor: USAID/FrontlineSMS Outcome Data: N	Pilot study using Standard Day Method to deliver fertility information to women via mobile phone technology. Allows women to know which days they are able to get pregnant with linkage to family planning services.	×		×	×	×	

SMS Alerts for Infant Vaccinations [87] India Sponsor: Information Kerala Mission Outcome Data: N	SMS system to inform parents on vaccination details and dates is linked to health center kiosks.	×		×			×

Target group: providers and mothers							

Smartphone-based fetal monitors [47] Australia Sponsor: Microsoft Outcome Data: N	Free software downloaded to mobile phones allows women to track fetal activity (kicks, heartbeat) through low-cost fetal monitors and transmit the data to midwives and obstetricians at urban and regional health centers.			×			×

MAPEDIR [88] Madhya Pradesh, India Sponsor: UNICEF Outcome Data: N	Investigative tool for why women are dying in pregnancy linked with 24-hour Janani Suraksha Obstetrician Helpline.	×		×	×	×	

SMS prenatal support for pregnant women [89] Bangkok, Thailand Siriraj Hospital/Mahidol University Outcome Data: Y	Prenatal health support via text messaging for women receiving care at a clinic.					×	

JiVita [90] Bangladesh Sponsor: Johns Hopkins University Outcome Data: Y	Most recent version of the JiVita program uses mobile phone technology to link mothers and families to sources of care and decrease pregnancy-related complications.	×		×		×	×

Mobile phones to coordinate ambulance [54] Gambia Sponsor: WHO Outcome Data: Y	Emergency ambulance service links the community with hospitals. Traditional birth attendants and village health workers were equipped with mobile phones to coordinate care. Pregnant women were high utilizers of the emergency ambulance.					×	×

Lady Health Workers [91] Pakistan GSMA/Mobilink/UNFPA/ Ministry of Health Outcome Data: Y	Linking mothers with Lady Health Workers through the use of Mobilink mobile phone technology to provide emergency care to rural areas.			×		×	×

D-tree [37] Tanzania UNICEF Outcome Data: NA	Software allows screening, examination, counseling, and treatment of malnutrition through calculations of target weights and measurements. Supports communication between health workers and mothers with information available via mobile phone.	×		×	×		×

MoTeCH [30] Ghana Mailman School of Public Health/Grameen/Gates Foundation Outcome Data: Y	Mobile Midwives provides expecting/new mothers accurate health information and reminders of upcoming clinic checkups. Option of text or spoken word, and language choices. Nurse Support allows nurses to search for patients with upcoming appointment dates and patient updates, such as new defaulters or recent deliveries. Patient information can be entered into mobile phones and used to generate reports and coordinate care.	×		×	×		×
